# Development and Evaluation of Paclitaxel and Curcumin Dry Powder for Inhalation Lung Cancer Treatment

**DOI:** 10.3390/pharmaceutics13010009

**Published:** 2020-12-22

**Authors:** Wing-Hin Lee, Ching-Yee Loo, Daniela Traini, Paul M. Young

**Affiliations:** 1 Faculty of Pharmacy and Health Sciences, Royal College of Medicine Perak, Universiti Kuala Lumpur (RCMP UniKL), Ipoh 30450, Perak, Malaysia; cyloo@unikl.edu.my; 2 Respiratory Technology, Woolcock Institute of Medical Research, Discipline of Pharmacology, Faculty of Medicine and Health, University of Sydney, Sydney, NSW 2037, Australia; daniela.traini@sydney.edu.au

**Keywords:** lung cancer, paclitaxel, curcumin, dry powder inhalation

## Abstract

Despite the effort to develop efficient targeted drug delivery for lung cancer treatment, the outcome remains unsatisfactory with a survival rate of 15% after 5 years of diagnosis. Inhalation formulation is an ideal alternative that could ensure the direct deposition of chemotherapeutics to the lungs. However, the design of an inhalable formulation that could simultaneously achieve a high local chemotherapeutic dose to the solid tumor and exert low pulmonary toxicities is a challenge, as the presence of 10–30% of chemotherapeutics in the lung is sufficient to induce toxicity. Therefore, this study aimed to develop a simple dry powder inhalation (DPI) formulation containing a model chemotherapeutic agent (paclitaxel, PTX) and a natural antioxidant (curcumin, CUR) that acts to protect healthy lung cells from injury during direct lung delivery. The co-jet-milling of CUR and PTX resulted in formulations with suitable aerosol performance, as indicated in the high fine particle fractions (FPF) (>60%) and adequate mass median aerodynamic diameter (MMAD). The CUR/PTX combination showed a more potent cytotoxic effect against lung cancer cells. This is evident from the induction of apoptosis/necrotic cell death and G2/M cell cycle arrests in both A549 and Calu-3 cells. The increased intracellular ROS, mitochondrial depolarization and reduced ATP content in A549 and Calu-3 cells indicated that the actions of CUR and PTX were associated with mitochondrial oxidative stress. Interestingly, the presence of CUR is crucial to neutralize the cytotoxic effects of PTX against healthy cells (Beas-2B), and this is dose-dependent. This study presents a simple approach to formulating an effective DPI formulation with preferential cytotoxicity towards lung cancer.

## 1. Introduction

Lung cancer contributes to the highest mortality rate among all common cancers, with 1.76 million deaths out of 9.6 million death caused by cancer in 2019 [1]. The majority of lung cancer patients are diagnosed at an advanced stage in which surgical resection is no longer feasible. Although these patients are treated with platinum-based chemotherapy or radiation, the outcome is lackluster with a survival rate of merely 15% after 5 years of diagnosis [2]. It is believed that the lack of effective localized drug delivery mechanisms and the poor understanding of the complexity of the tumor microenvironment in lung cancer progression contribute to ineffective diagnosis and therapy [3]. Current treatment with non-specific and non-selective chemotherapeutic agents delivered via infusion, injection and/or oral route typically result in severe systemic toxicities. As the concentrations of the drug that reach the solid tumor in the lung are usually below the therapeutic range, higher and more frequent dosing are needed which ultimately leads to various systemic complications and the development of irreversible multi-drug-resistant cancer cells. The manifestation of systemic toxicities often requires treatments to be interrupted to allow the recovery of healthy cells [4]. However, this leads to the re-growth of cancer cells and possible metastasis to other organs. Owing to the complexity of multidrug resistance in the tumor, combination therapy in which each drug has a different mechanism of action is a more effective strategy to inhibit the progression of lung cancer. For example, the combination of docetaxel and vandetanib improved progression-free survival compared to mono docetaxel treatment in non-small-cell lung cancer [5].

The inhalation route is an increasingly popular alternative route for the delivery of chemotherapeutic agents to ensure effective drug localization in lung tumors. Pulmonary drug delivery is associated with higher local drug concentration, as the drug could bypass the intracellular and extracellular drug-metabolizing enzyme activity in the gastrointestinal tract and liver [6]. However, this delivery route for aerosolized chemotherapy has had very little success, with a clinical trial unable to progress beyond phase II, although feasibility and safety data demonstrated that aerosolized chemotherapeutics are well-tolerated [7]. The clinical findings of nebulized formulations observed that a high local chemotherapeutic dose to the solid tumor is hard to achieve, with the poor aqueous solubility of many chemotherapeutic agents resulting in a low drug concentration in the nebulized formulation. In addition, designing a formulation contributing to low pulmonary toxicities following high local delivery and drug residency is a challenge, since 10–30% of chemotherapeutics in the lung is adequate to induce toxicity. Dry powder inhalers (DPIs) present many advantages over liquid nebulizers. DPI-based formulations are solid-state and thus more suitable for poorly soluble chemotherapeutic drugs. In addition, the administration time of DPI is short in comparison to the longer time needed for nebulizer administration [8].

To achieve a clinical therapeutic dose in the lung coupled with subsequent low pulmonary toxicities, a simple DPI approach using a combination of drugs is designed for this study. A conventional chemotherapeutic agent is used in combination with a natural antioxidant that could protect healthy lung cells from injury. We chose paclitaxel (PTX) as the model chemotherapeutic agent for this study as it is widely used for lung cancer treatment. PTX acts to promote the polymerization of tubulin, which induces cell death by disrupting the dynamics of the normal microtubule that is required for cell division and important interphase processes. In addition, PTX induces cell arrest in the G2/M phase of the cell cycle [9]. Like most typical chemotherapeutics, PTX is associated with various toxic effects such as neurotoxicity, hematological toxicities and hypersensitivity reactions [10]. To neutralize the PTX toxicities in healthy cells, we propose to incorporate curcumin (CUR) into the DPI formulations. CUR is a yellow-colored polyphenolic compound derived from the rhizome of *Curcuma longa* that has been demonstrated to exert preferential toxicities towards cancer cells. This is supported by findings from Caroll et al., whereby CUR displays chemopreventive activities in humans [11]. Substantial evidence also revealed the radioprotective nature of CUR against several normal tissues [12]. In addition, CUR is known to possess a range of pharmacological effects, such as antioxidant, anti-inflammatory and anti-cancer activities. Therefore, the combined delivery of PTX and CUR via inhalation is postulated to exert stronger anti-cancer activities while protecting healthy lung cells from extensive cell damage and death. Despite this, the application of CUR as an anti-cancer agent is hampered owing to its poor water solubility (partition coefficient value, log *P* of 3), poor stability in alkaline conditions and extensive hepatic metabolisms. Several published works on the engineering of nano-DPI for inhalation have been published. They are mainly based on the approach of developing the formulation in the form of nano-in-microparticles and reversible aggregates, whereby the former is held together by a micron-sized carrier while the latter is agglomerated owing to the action of Van der Waals interactions [13]. In this work, a simple DPI formulation is chosen, whereby the PTX and CUR blends were first mixed homogenously and co-jet-milled to form microparticles suitable for inhalation. It should be highlighted that this study is a proof of concept that the incorporation of a natural antioxidant compound enhances both the protective effects against healthy lung cells as well as anti-cancer activities. Therefore, in the present study, we aimed to evaluate the aerosol performance of a DPI combination formulation of PTX and CUR and its anti-cancer properties in vitro in different immortalized human lung adenocarcinoma (Calu-3) and human lung carcinomas (A549) cell lines. Besides this, the protective effects of these combination formulations against healthy lung cells (Beas-2B) were also investigated. 

## 2. Material and Methods

### 2.1. Materials

Paclitaxel (PTX) was supplied by Wuhan Demeikai Biotechnology Co, Ltd. (Wuhan, China). Curcumin (CUR), dimethylsulfoxide (DMSO), trifluoroacetic acid, non-essential amino acids, Dulbecco’s Modified Eagle Medium and DMEM: F12 medium were obtained from Sigma-Aldrich (Sydney, Australia). Cell culture reagents such as trypsin-EDTA solution, _L_-glutamine solution (200 mM) and fetal bovine serum (FBS) were obtained from Invitrogen (Sydney, Australia). CellTiter 96 MTS was purchased from Promega Co, Madison, WI, USA. Analytical grade absolute ethanol and the acetonitrile used were supplied from Fronine, Sydney, Australia.

### 2.2. DPI Blends Manufacture

PTX and CUR powders were mixed and shaken in a steel container (Hexagon Ltd., Model Alphie-03) for 60 min at 32 rpm. In this study, three different combinations of PTX and CUR blend were prepared: (a) blend consisting of 75% CUR (*w*/*w*) and 25% PTX (*w*/*w*); (b) blend consisting of 50% CUR (*w*/*w*) and 50% PTX (*w*/*w*), and (c) blend consisting of 25% CUR (*w*/*w*) and 75% PTX (*w*/*w*). Unless otherwise stated, the three formulations are referred to correspondingly as 75CUR:25PTX, 50CUR:50PTX and 25CUR:75PTX hereafter. The combination formulations are also referred to as CUR/PTX formulation in general throughout the manuscript.

Each blend was co-milled using a jet-mill (Labo Mill Micronization Equipment FPS 0447, Fiorenzuola d’Arda, Italy), at a feeding rate, injection pressure and feeding pressure of 1 g/min, 7 bars and 7 bars, respectively. The resultant samples were stored in a chamber with controlled temperature and humidity (50% RH, 25° C) until further analysis. Besides this, the micronization of CUR alone and PTX alone was also conducted using the same milling process.

### 2.3. Particle Characterization 

#### 2.3.1. Particle Size Measurement

The size distribution of both jet-milled powders was measured using laser diffraction with a dry feed cell (Malvern Mastersizer 3000, Instruments Ltd., Malvern, UK). Briefly, 5 mg of sample was dispersed in air with 4 bars pressure and measured at an obscuration of 5–15%, with a refractive index 1.53. All measurements were conducted in triplicate and the d_10_, d_50_ and d_90_ were recorded.

#### 2.3.2. Scanning Electron Microscopy

Visualization of particles before and after jet-milling was conducted using a scanning electron microscope (SEM) at 15 keV (JEOL-JCM 6000 NeoScope Benchtop SEM, Tokyo, Japan) under a range of magnifications. Samples were placed onto SEM stubs, coated with gold (approximate thickness of 15 nm) and viewed under SEM.

#### 2.3.3. Blend Homogeneity

The drug content uniformity of samples for co-jet-milled PTX and CUR samples in different ratios was measured according to the British Pharmacopoeia Volume V Appendix XII C. 3. Test B [14]. Briefly, the samples were placed and dispersed evenly over wax paper and ten random samplings (10 ± 1 mg) were conducted. This was followed by dissolving the sampled powders in 70:30 *v*/*v* acetonitrile:water, filtered with a 0.22 μm nylon filter and assayed using high-performance liquid chromatography (HPLC). The resultant concentration of CUR and PTX for each sample was presented as a percentage with respect to the theoretical amount of drugs in the sample. As per requirement by the pharmacopoeia, a blend sample with the range of 85–115% of the nominal dose is considered homogenous [14].

#### 2.3.4. In Vitro Aerosol Performance

A Mono-dose RS01 device (Plastiape, SPA, Osnago, Italy) with high resistance (HR) was used to deliver DPI formulations of CUR only, PTX only and CUR/PTX combinations with varying ratios. The aerodynamic particle size distributions of different formulations were assessed using Next Generation Impactor (NGI, Apparatus 5, USP Test chapter < 601>, Copley Scientific, Nottingham, UK). The cut-off diameters corresponding to each stage at a flow rate of 60 L/min are as follows: stage 1 (11.72 µm), stage 2 (6.4 µm), stage 3 (3.99 µm), stage 4 (2.3µm), stage 5 (1.35 µm), stage 6 (0.83 µm) and stage 7 (0.54 µm). Approximately 10 ± 1 mg of DPI was weighed into a size 3 gelatin capsule, placed into the RS01 device and aerosolized at 60 L/min for 4s using a calibrated flow meter (Model 4040, TSI Model Instruments, Aachen, Germany). For this study, only 1 actuation was used for all formulations. After actuation, each component of NGI was rinsed separately with 70:30 *v*/*v* acetonitrile:water and assayed using HPLC. The experiments were done in triplicate. The fine particle fraction (FPF) of the aerosolized formulation is defined as the percentage of particles ≤5 μm of emitted dose. The fine particle emitted dose (FPD) refers to the mass of particles ≤5 μm of the emitted dose. The regression of the log-linear of stage-size verses cumulative stage deposition was plotted to calculate mass median aerodynamic diameter (MMAD) and FPF. The recovered dose is defined as the sum of the CUR/PTX mass assayed by HPLC on all stages in a single run. As per requirement, the total mass recovered should be in the range of 85–115% of a nominal dose. The emitted dose (ED) is the sum of all mass collected from throat and all stages from 1 to 7. The emitted fraction (EF) is expressed as the mass percentage of CUR/PTX that exited the inhaler with respect to the recovered dose.

#### 2.3.5. Drug Dissolution

The dissolution behaviors of DPI formulations of CUR only, PTX only and CUR/PTX combinations were investigated using a USP paddle apparatus with a rotational speed of 50 rpm (DIS 8000, Copley Scientific, Nottingham, UK). The medium used was phosphate buffer at pH of 7.4 containing 1% Tween 80. The temperature was maintained at 37 ± 0.5 °C. Three independent replicates were conducted for each sample. In total, 30 mg of samples were placed into the apparatus and 2 mL of samples were withdrawn at specific time intervals. Fresh dissolution medium was immediately replaced into each vessel. Samples were filtered with a 0.45 μm filter membrane and analyzed with HPLC.

#### 2.3.6. High-Performance Liquid Chromatography (HPLC) Analysis

The quantification of PTX and CUR was performed using HPLC (Shimadzu, Sydney, Australia). The mobile phase consisting of a mixture of 70:30 *v*/*v* acetonitrile:water and 0.1% *v*/*v* trifluoroacetic acid was used for HPLC analysis and operated at the flow rate 1.5 mL/min with a photodiode array detector (PDA) at 25 °C using a C18 column (Nova-Pak, 150 × 4.6 mm, Waters, MA, USA). The injection volume for each sample was 100 µL and the concentrations of the drugs were analyzed via a UV wavelength of 430 nm and 227 nm for CUR and PTX, respectively. The retention time for CUR was 2.6 min and for PTX was 2.7 min. The limit of detection (LOD) values for CUR and PTX were 10 ng/mL and 10 ng/mL, respectively.

### 2.4. In Vitro Cell Evaluation

#### 2.4.1. Cell Culture

Calu-3 and Beas-2B were cultured separately in Dulbecco’s Modified Eagle Medium (DMEM): Ham’s (F12) medium supplemented with 10% Fetal Bovine Serum (FBS) and 1% non-essential amino acids. All cell lines were bought from American Type Culture Collection (ATCC) (Manassas, VA, USA). A549 cells was cultured in DMEM medium containing 10% FBS. All cells were grown in a humidified incubator at 37 °C with 5% CO_2_ and 95% humidity until they reached confluence. Every 2–3 days the medium was changed, and cells were passaged according to ATCC guidelines.

#### 2.4.2. Cell Cytotoxicity Assay

The cytotoxicity of the drug formulations towards Calu-3, A549 and Beas-2B cells was evaluated using the MTS assay. Briefly, approximately 50,000 cells per well were seeded onto 96 well plates and incubated overnight at 37 °C in 5% CO_2_ and 95% RH to allow cell adherence. Cells were then treated with CUR alone, PTX alone and CUR/PTX combinations at varying final concentrations (0 µM, 0.315 µM, 0.625 µM, 1.25 µM, 2.5 µM, 5.0 µM, 10.0 µM, 20.0 µM, 30.0 µM, 40.0 µM, 50.0 µM, 60.0 µM, 70.0 µM, 80.0 µM, 90.0 µM and 100 µM) for 72 h. The stock solutions were prepared by dissolving the samples in dimethyl sulfoxide (DMSO) and the final concentration of DSMO in the cell culture medium was maintained below 1% (*v*/*v*). This was followed by the addition of MTS reagent (20 µL) and it was incubated for 4 h at 37 °C (5% CO_2_ and 95% RH) before measurement of the absorbance at 490 nm (SpectraMax M2; Molecular devices, Sydney, Australia). The cell viability (%) was determined by comparing the absorbance of treated cells to that of untreated cells (control). The IC_50_ values were determined using GraphPad Prism 6 software by plotting cell viability (%) against the drug concentrations (µM). Each concentration was performed in triplicate. 

#### 2.4.3. Cellular Uptake

The cellular uptake of drugs in different lung cells (A549, Calu-3 and Beas-2B) was measured by quantifying the total drug concentration present inside the cells. Briefly, 5 × 10^4^ cells per well were inoculated onto 24-well plates and incubated overnight at 37 °C. The cells were then treated with different formulations of PTX/CUR at 10 µM total concentrations and incubated for another 24 h at 37 °C. At a predetermined time point, cells were washed with PBS (3 times), lysed with cell lytic reagent and re-suspended in 1 mL of solvent consisting of 70:30 *v*/*v* acetonitrile:water. After centrifugation at 13,000 rpm at 4 °C for 10 min, the clear supernatant was collected and assayed with HPLC to measure corresponding PTX and CUR concentrations in cells. The cellular uptake was expressed as total drugs (ng)/total protein (µg). Three independent experiments were conducted for this purpose.

#### 2.4.4. Cell Apoptosis

The abilities of PTX and CUR formulations to promote apoptosis in A549, Calu-3 and Beas-2B were measured by using APC Annexin V and 7AAD according to manufacturer’s instructions (BD Biosciences, Sydney, Australia). Briefly, approximately 5 × 10^5^ cells were seeded onto the 35 mm tissue culture plate and incubated for 24 h, followed by the different drug formulations treatment at 10 µM for another 48 h. Cells were washed twice with cold PBS (2 times), detached by trypsin and harvested by centrifugation before being re-suspended in 100 μL of 1× binding buffer. This was followed by adding 5 μL of APC annexin V and 5 μL of 7AAD, and it was then incubated at room temperature in dark conditions for 20 min. The apoptotic level of cells was measured based on the fluorescence intensity of APC annexin V and 7AAD by using flow cytometry (BD Biosciences, Sydney, Australia) with at least 10,000 events. The experiments were conducted in triplicate independently.

#### 2.4.5. Cell Cycle Analysis

A549, Calu-3 and Beas-2B cells (5 × 10^5^ cell per well) were separately seeded into 35 mm tissue culture plates and incubated for 24 h. Then, the cells were further treated with 10 µM of different formulations of PTX and CUR for 48 h. After treatment, cells were washed twice with PBS, trypsinized and fixed with ice-cold 70% ethanol. After fixation, the cells were washed with PBS and re-suspended into 100 µL PBS containing 0.1% of Triton X-100 and 10 mg/mL of RNase for 10 min. Then, 1 μL of PI (1 mg/mL) was added and incubated for 20 min in dark conditions at room temperature. The fractions of fluorescent events from the interaction between PI and DNA after treatment with different formulations were analyzed using flow cytometry (BD Biosciences, Sydney, Australia) and at least 10,000 events were counted. Three independent experiments were conducted for this purpose.

#### 2.4.6. ATP Production

To evaluate the ATP level of A549 and Calu-3 with the PTX and CUR formulations treatment (10 µM total concentration), 1 × 10^5^/mL of stock cell solution was prepared in complete DMEM medium without phenol and 100 µL of cell suspension was seeded onto a 96-well plate and incubated for overnight. Next, the cells were replaced with 100 µL of medium that contained a different combination of PTX and CUR, and incubated for another 6 h. The intracellular ATP content level was measured by luminescent ATP detection assay kit (Abcam, Sydney, Australia) according to the protocol provided by the company. The density of luminescence was measured by the FLUOstar^®^ Omega (BMG LABTECH, Ortenberg, Germany) that was equipped with a luminescence detector. Three independent experiments were conducted for this purpose.

#### 2.4.7. Mitochondrial Membrane Potential

The changes in mitochondrial membrane potential (MMP) in different lung cells (A549, Calu-3 and Beas-2B) when treated with different formulations of PTX and CUR (10 µM total concentration) were measured using the JC-10 mitochondrial membrane potential assay kit (MAK159), Sigma-Aldrich (Sydney, Australia). Before the treatment, approximately 5 × 10^4^ cells per well were inoculated into a black 96-well plate and incubated overnight. This was followed with a treatment of CUR alone, PTX alone and a combination of CUR/PTX at varying ratios for 24 h. Cells without treatment and medium were used as a control in the study. After treatment, cells were washed with PBS twice and this was followed by staining with JC-10 dye as per the protocol supplied by the manufacturer. The intensity of fluorescence was measured by the (SpectraMax M2; Molecular devices, Sydney, Australia) microplate reader at λ_ex_ = 490/λ_em_ = 525 nm for green fluorescence and λ_ex_ = 540/λ_em_ = 590 nm for red fluorescence. The ratio between red and green fluorescence over control was taken as a percentage of MMP depletion. Three independent experiments were conducted for this purpose.

#### 2.4.8. Reactive Oxygen Species Measurement

The intracellular reactive oxygen species (ROS) levels of A549, Calu-3 and Beas-2B cells treated with CUR alone, PTX alone and a combination of CUR/PTX at varying ratios (10 µM total concentration) were measured based on the production of fluorescent dichlorofluorescein (DCF). For this purpose, briefly, 100 µL of cell suspension (5 × 10^4^ cells per well) was seeded onto the 96-well plate. After overnight incubation, the cells medium was replaced with fresh medium (100 µL) containing 5 µM of 2′,7′-dichlorofluorescein diacetate (DCFH-DA) and this was incubated for 30 min in dark conditions. Next, the media containing DCFH-DA were removed and replaced with the fresh medium containing drug formulations. After 30 min of treatment, cell-free supernatant was transferred into a 96-well plate for fluorescence measurement (SpectraMax M2; Molecular devices, Sydney, Australia) at 485 nm (excitation) and 520 nm (emission). In this experiment, _L_-ascorbic acid (1 mM) and H_2_O_2_ (0.03%) were used as negative and positive controls, respectively. Three independent experiments were conducted for this purpose.

### 2.5. Statistical Analysis

Data were expressed as mean ± standard deviation (SD), in which each experiment was done in triplicate to confirm reproducibility. Data were analyzed using two-way analysis of variance (ANOVA). The statistical significance of data was determined by Tukey test, with *p* < 0.05 considered statistically significant.

## 3. Results and Discussion

### 3.1. Particle Morphology and Size Distribution

**Figure 1:**
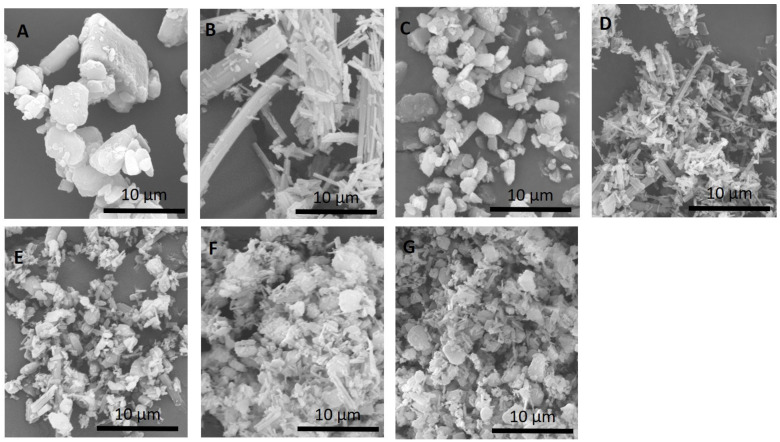
SEM images of (**A**) raw CUR, (**B**) raw PTX, (**C**) jet-milled CUR, (**D**) jet-milled PTX and the combination of milled CUR/PTX at different ratios ((**E**) 25CUR:75PTX, (**F**) 50CUR:50PTX and (**G**) 75CUR:25PTX). It should be noted that 25CUR:75PTX refers to the combination with the ratio of CUR to PTX at 25:75. CUR: curcumin; PTX: paclitaxel.

**Figure 2:**
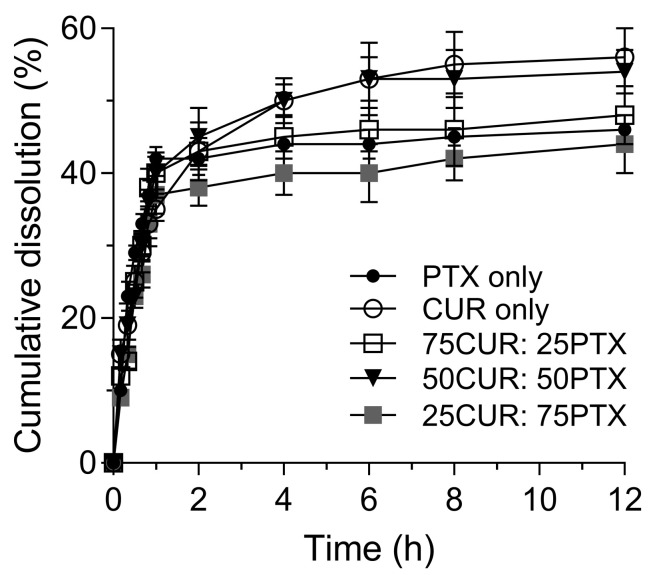
Comparative dissolution profile of PTX, CUR and CUR/PTX combination in phosphate buffer at a pH of 7.4 containing 1% Tween 80.

Figure 1 shows the changes in particle size and morphology of samples before and after the milling process. Before the milling process (Figure 1A), CUR particles exhibited a crystalline structure that is heterogeneous with a size of ~22 µm. No significant changes in particle morphology could be observed for CUR after the milling. A reduction in the size and length of CUR crystals is observed (˂5 µm) after milling (Figure 1C). Meanwhile, the PTX particles (Figure 1B) before milling were elongated needle-like crystals of ~42 µm in length. A heterogeneous mixture of particle size of PTX was observed after milling, and significant agglomerations of particles are also noted (Figure 1D). The physical mixing of jet-milled PTX and CUR at varying ratios revealed a homogenously blended formulation (Figure 1E–G). Laser diffraction analyses showed that all the formulations have similar size distributions, in which the d_90_ and d_10_ ranged between 4.9 and 5.7 µm and 0.9 and 1.5 µm, respectively (Table 1). In the blend homogeneity experiment, it is demonstrated that the co-jet-milled samples are within the acceptable range, whereby not more than one individual sample was outside the limits of 85 to 115% of the average content. As observed by Lau et al., the jet-milling procedures resulted in the simultaneous mixing and micronization of samples, which led to a homogeneous distribution of drugs [15]. The results of dissolution studies of CUR alone, PTX alone and the combination of CUR/PTX at varying ratios under controlled conditions are illustrated in Figure 2. The dissolutions of CUR alone and PTX alone exhibited similar patterns, whereby approximately 50% of the drugs were dissolved after 12 h. Our data accord with other published findings under similar controlled conditions [16,17]. In addition, the dissolutions of combination formulations also followed similar trend. No significant differences in the amounts of dissolved drugs were observed for all tested formulations.

### 3.2. In Vitro Aerosol Performance

**Figure 3:**
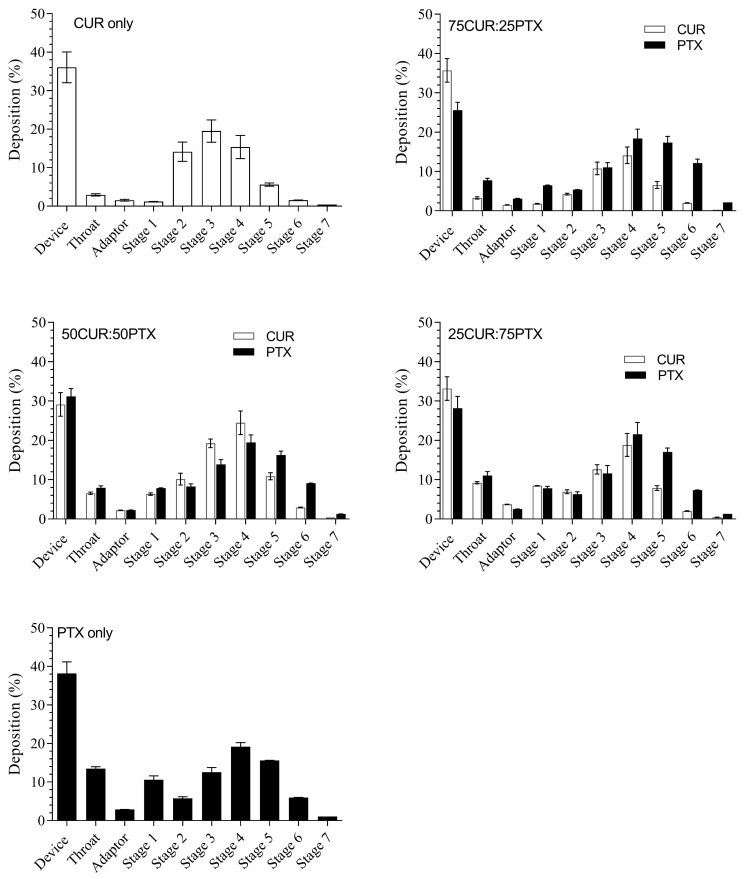
In vitro aerosol deposition of the milled CUR, PTX and combination formulations using NGI at a flow rate of 60 L/min with HR RS01 DPI device. Data represent mean ±SD (n = 3). Black bar denotes PTX and white bar denotes CUR. Data are expressed as mean ± SD of three replicates.

The in vitro evaluation of the aerosol deposition patterns of the DPI formulations using the NGI is shown in Figure 3. The deposition in the device ranged from 30 to 40% for all samples in general (Figure 3). This could be due to the cohesive nature of the CUR/PTX microparticles, which promoted agglomeration and hence poor dispersion. No significant changes in the deposition pattern of each formulation (CUR and PTX) in throat, adaptor and NGI stages were observed, which suggested that the combination formulations were homogeneously mixed. Minimal deposition in the throat and adaptor was observed for CUR alone using high-resistance devices. The effect is more pronounced when the ratio of PTX is increased; for instance, the CUR deposition in the throat increased from 2.9 ± 0.3% (CUR alone) to 9.5 ± 0.3% (25CUR:75PTX) (Figure 3). An increase in the surface area of milled particles coupled with the irregular needle-like shape might have increased the electrostatic interactions and cohesiveness between PTX and CUR. Therefore, this reduced the de-agglomerations of particles after fluidization and increased the depositions in the throat and adaptor. When the high-resistance RS01 device was used, the FFP and MMAD for aerosolized CUR alone were 69.5 ± 1.0% and 3.00 ± 0.05 µm, respectively (Table 2). No significant differences in the aerosol characteristics for CUR for the low-resistance RS01device were observed (*p <* 0.05, results not shown). Meanwhile, the FFP and MMAD for aerosolized PTX alone were 60.4 ± 1.5% and 2.85 ± 0.10 µm, respectively (Table 2). For all combination formulations, the FPF values ranged between 60 and 70%, with 50% of the population below 3.12 µm in size (Table 2). The ED values of CUR fractions for the 100CUR, 75CUR:25PTX, 50CUR:50PTX and 25CUR:75PTX are 6392.6 ± 254.6 µg, 4887.6 ± 195.0 µg, 3557.0 ± 150.0 µg and 1600.0 ± 40.0 µg, respectively (Table 3). Meanwhile, for PTX, the corresponding ED values for 100PTX, 75CUR:25PTX, 50CUR:50PTX and 25CUR:75PTX are 6044.2 ± 184.2 µg, 1755.8 ± 37.0 µg, 3429.4 ± 140.4 µg and 5263.0 ± 160.0 µg, respectively.

### 3.3. Biological Activities of CUR/PTX Formulations

Table 3 shows the MTS cytotoxicity evaluation of the PTX and CUR combination formulation against healthy lung and cancer cell lines. The IC_50_ value of PTX against healthy lung cells (Beas-2B) is 8.1 ± 0.5 µM and is the most cytotoxic compared to other formulations. This finding is in accord with other published works which described the dose-dependent cytotoxicity behavior of PTX against Beas-2B. An almost three-fold reduction in cell survival was noted (28.1% to 9.7%) as the PTX concentration was increased from 10 µg/mL to 80 µg/mL [18]. CUR exerted negligible cytotoxic effects against Beas-2B as the cells’ viability remained >90% against all tested concentrations (Table 3). Several studies have demonstrated that CUR displayed a preferential affinity towards cancer cells compared to healthy cells, which could be associated with the higher internalization of CUR into cancer cells [19,20]. We previously showed that CUR was not internalized effectively by healthy cells (Beas-2B and HOf cells), while it was internalized and distributed in the membrane, cytoplasm and nucleus of cancer cells [21,22]. Apart from cell uptake, Syng-ai et al. reported that CUR induced higher cell apoptotic and death populations in cancer cells via generation of superoxide and ROS [23]. Consistent with other findings, the authors showed that CUR did not induce superoxide formation in normal rat hepatocytes, and therefore induced negligible cell death [23].

Interestingly, the presence of CUR seems to neutralize the cytotoxic effect of PTX, whereby the combinations of CUR and PTX at the ratios of 75:25 and 50:50 showed undetectable IC_50_ values at tested concentrations (Table 3). The incorporation of 25% CUR into the PTX formulation significantly increased the IC_50_ value from 8.1 ± 0.5 µM to 20.3 ± 1.7 µM. Many studies observed an increase in oxidative stress and pro-inflammatory events following the exposure of cells to PTX in both healthy and cancer cells [24,25]. In a study by Ibrahim et al., the co-delivery of an antioxidant compound (sodium-*R*-lipoate) with chemotherapeutic agents reduced the cell death from 68% to 10% [4]. In our study, it is possible that the cytoprotective role of CUR was due to its carbon–carbon double bonds, diketo group, and phenyl rings with hydroxyl and methoxy substituents that contribute to antioxidant and anti-inflammatory behavior [26]. As a natural antioxidant, it is believed that CUR scavenges free radicals and endogenous oxidizing active substances that are induced in cells grown in the presence of PTX. By positioning CUR within the cell membrane, the oxidative damage caused by peroxidation could be avoided [26]. CUR is also known to increase intracellular glutathione levels by maintaining the activity of histone acetyltransferase, thus reducing damage during oxidative stress [27]. The protective effect of CUR is linked to the reduction of apoptosis and oxidative stress via the activation of the Nrf2 signaling pathway in cells, which induces the expression of antioxidant enzymes and detoxification enzymes (i.e., heme oxygenase-1 and superoxide dismutase) [28,29]. In another study, CUR mitigated the oxidative stress in H_2_O_2_-induced-neurotoxicity PC12 cells via the suppression of mitogen-activated protein kinase (MAPK) and AKT pathways, thereby reducing apoptosis and cell death [30].

Among the different tested formulations, the cytotoxicity effects of PTX alone against both lung cancer cell lines (A549 and Calu-3) were the lowest, as reflected in the highest IC_50_ values (>40 µM). In comparison, the IC_50_ values of CUR alone against A549 and Calu-3 are 26.3 ± 2.9 and 30.3 ± 2.5 µM, respectively. The presence of CUR increases the sensitivity of cancer cells towards PTX. Irrespective of the cancer cell lines, the combination of CUR and PTX at the ratio of 75:25 was the most cytotoxic, whereby the IC_50_ values for A549 and Calu-3 were 18.9 ± 3.5 and 22.9 ± 1.8 µM, respectively. The effectiveness of these formulation against A549 cells followed the decreasing order of 75 CUR:25 PTX (18.9 ± 3.5 µM) > 50 CUR:50 PTX (22.1 ± 0.5 µM) > CUR only (26.3 ± 2.9 µM) > 25 CUR:75 PTX (32.5 ± 2.3 µM) > PTX only (47.5 ± 2.6 µM). A similar trend was observed for Calu-3 cells lines in which a combination of CUR and PTX at a ratio of 25:75 was the least effective in killing cancer cells among the drug combination formulations.

**Figure 4:**
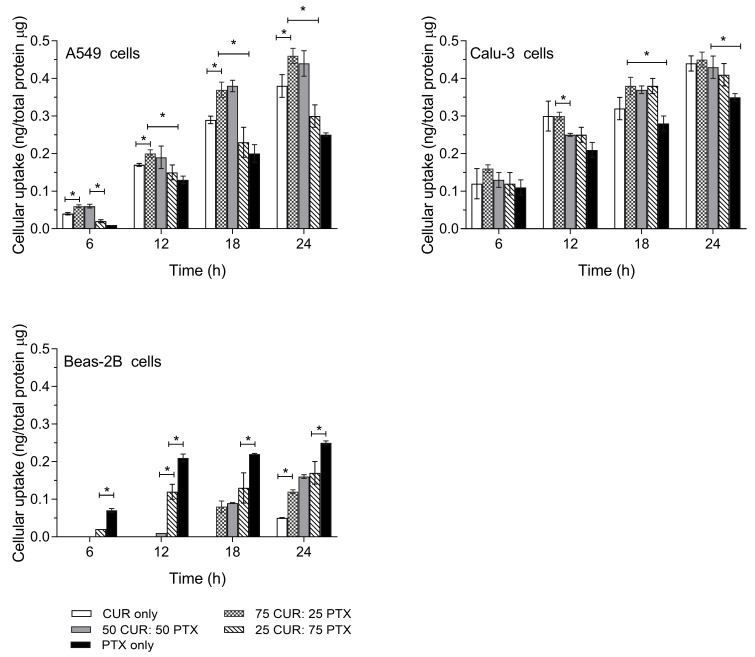
The cellular uptakes of single and combination formulations in A549, Calu-3 and Beas-2B as a function against time. Data are expressed as mean ± SD of three replicates. * Significantly different at *p* < 0.05 (Tukey post hoc test).

The quantitative cellular uptake of PTX, CUR and the combination of CUR/PTX as a function of time is shown in Figure 4. It was demonstrated that the cellular uptake of PTX alone for both cancer cell lines was the lowest and was statistically significant compared to other formulations containing CUR. For A549 cells, the combinations of CUR and PTX at the ratios of 75:25 and 50:50 demonstrated the highest cellular uptakes followed by the CUR alone and 25CUR:75PTX formulations. This trend was observed at all the tested time points. The cellular uptakes for 75CUR:25PTX and 50CUR:50PTX formulations at 24 h were not statistically significant at 0.46 and 0.44 ng/mg total protein, respectively. However, the drug internalization was significantly higher (*p* < 0.05) than other formulations. In comparison, the cellular uptakes for 25CUR:75PTX, CUR alone and PTX alone were 0.30, 0.38 and 0.25 ng/µg total protein, respectively (Figure 4). As calculated with Tukey post-hoc test, the p value for the cellular uptake of 75CUR:25PTX and 50CUR:50PTX was *p* = 0.43. Meanwhile, the p values for 75CUR:25PTX compared with 25CUR:75PTX, CUR alone and PTX alone were *p* = 0.001, 0.018 and < 0.001, respectively. For Calu-3, there were no statistical differences in the cellular uptake for all the samples at 6 h. The concentrations of the internalized drug ranged from 0.11 to 0.16 ng/µg total protein. As the cell uptake was prolonged to 24 h, it was noted that the cellular uptake of PTX was significantly lower compared to other formulations at 0.35 ng/mg total protein. The p values of PTX compared to 75CUR:25PTX, 50CUR:50PTX, 25CUR:75PTX and CUR alone were *p* < 0.0001, 0.0013, 0.0248 and 0.0003, respectively. At 24 h, the drug uptakes for formulations containing CUR ranged between 0.41 and 0.45 ng/µg total protein. A statistically higher PTX internalization into Beas-2B compared to all other formulations was noted, which supported the cytotoxic effect of PTX against healthy cells. In comparison, for CUR alone, the internalized drug content was only detected at 24 h.

**Figure 5:**
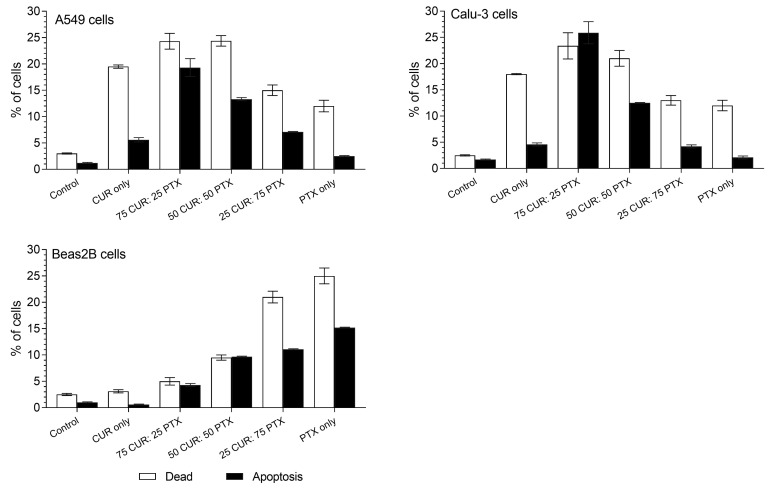
The effect of PTX, CUR and CUR/PTX combination on the apoptosis of A549, Calu-3 and Beas-2B cells. Control indicates untreated cells. Data are expressed as mean ± SD of three replicates.

The apoptotic event caused by the treatment of different lung cell lines with CUR alone, PTX alone and CUR/PTX was evaluated using APC annexin V and 7AAD staining (Figure 5). From the figure, it is evident that the treatment of lung cancer cells resulted in a significant increase in apoptotic and dead population, irrespective of the type of formulation. For untreated A549 cells, the % values of apoptotic and death populations were 1.2% and 3%, respectively. The treatment induced apoptosis in the following decreasing order: 75CUR:25PTX (19.3%) > 50CUR:50PTX (13.3%) > 25CUR:75PTX (7.1%) > CUR (5.6%) > PTX (2.5%). Formulations containing a higher proportion of CUR generally resulted in higher cell death populations. For instance, the treatment of A549 cells with 75CUR:25PTX and 50CUR:50PTX formulations resulted in approximately 24% cell death, which is twofold higher than PTX alone. Calu-3 cell lines followed a similar pattern, whereby a combination of CUR/PTX at a ratio of 75/25 showed the highest activities in both apoptosis (25.9%) and cell death (23.4%). As expected, the cell death and apoptosis of Calu-3 after treatment with PTX were the lowest, at 12% and 2.1%. Consistent with the MTS cytotoxicity and cellular uptake data, it is evident that the combination of CUR/PTX enhanced the apoptosis rate and cell death compared to CUR or PTX alone. Specifically, the cytotoxicity, cellular uptake and apoptosis rate were more pronounced when the combination had a higher CUR portion. Our data are in accord with a recent study by Calaf et al., whereby a combination of PTX and CUR synergistically induced higher apoptosis in cancer cells [31]. CUR is believed to sensitize the cancer cells to PTX through the down-regulation of the oncogene-related pathways, such as NF-ĸB [32]. The co-administration of CUR and PTX to mouse models of cervical cancer resulted in the deactivation of the NF-ĸB pathway (central regulator of over-expressions of anti-apoptotic genes in cancer cells), which was then accompanied with the activation and cleavages of various pro-caspases 3, 7, 8 and 9, and associated downstream genes [33]. The same observation was also reported by Kang et al., whereby the higher apoptosis in A549 cells following the treatment with CUR and carboplatin was due to the elevated expressions of caspase 3 and 9 [34]. The authors also demonstrated that other pro-apoptotic genes, such as p53, p21 and Bax, were also increased in A549 cells [34].

Besides this, the induction of apoptosis in Beas-2B further supported the MTS cytotoxicity data, whereby PTX alone caused a significant increase in cell death (25%) and apoptosis (15.2%) compared to untreated cells (2.5% death; 1% apoptotic). CUR alone did not exert a statistically significant increase in cell death and apoptosis compared to untreated cells. CUR seemingly acted to protect Beas-2B cells from significant cell damage, as is evident from the low cell death populations for the 75CUR:25PTX and 50CUR:50PTX formulations. Meanwhile, when the combination of CUR/PTX contained a higher proportion of PTX, the protective effect of CUR was diminished. Approximately 21% of the Beas-2B cell populations were dead, and 11.1% were in the apoptotic state (Figure 5).

**Figure 6:**
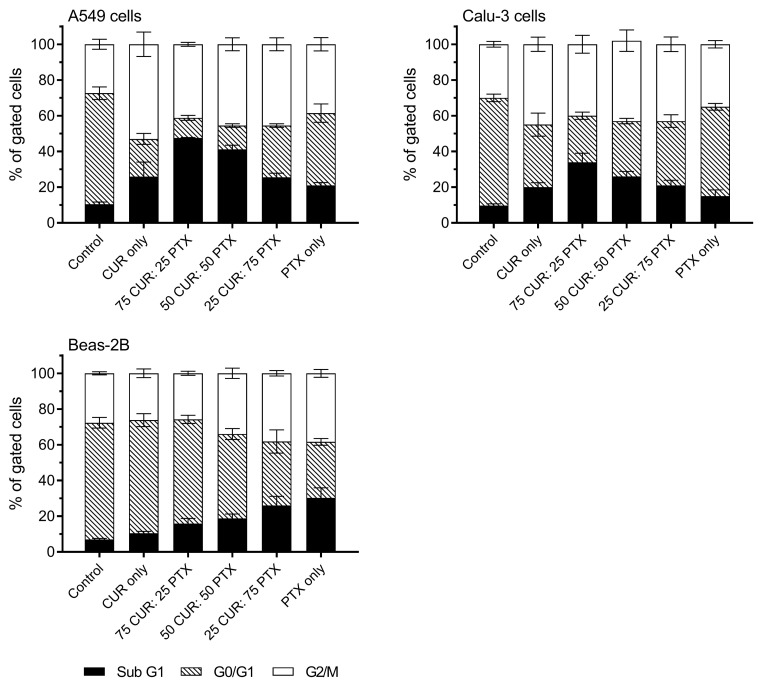
Cell cycle analysis of A549, Calu-3 and Beas-2B after treatment with a combination of CUR and PTX formulations. Control indicates untreated cells. Data are expressed as mean ± SD of three replicates.

The cell cycle analysis showed an increase in G2/M and Sub G1 in both Calu-3 and A549 cells for all treatments. The percent values of G2/M of untreated cells in the Calu-3 and A549 cells were 30.0 ± 1.6% and 27.4 ± 2.8%, respectively (Figure 6). The G2/M population in A549 cells after treatment was significantly higher compared to the untreated control, and the following order was observed: CUR alone (53.0 ± 6.8%, *p* < 0.0001) ˃ 50CUR:50 PTX (45.5 ± 3.0%, *p* < 0.0001) ˃ 25CUR:75PTX (43.5 ± 3.6%, *p* < 0.0001) ˃ 75CUR:25PTX (41.2 ± 1.1%, *p* < 0.0001) ˃ PTX only (38.5 ± 3.7%, *p* = 0.0231). All formulations also resulted in high A549 cell death, as indicated in the higher sub-G1 populations. It should be noted that the combination formulation with higher CUR ratio (i.e., 75% CUR and 50% CUR) was more effective in inducing cell deaths in both the A549 and Calu-3 cells. For Calu-3, a similar trend was observed in which all formulations induced higher G2/M arrest and sub-G1 populations. As for Beas-2B, it is interesting to note that the CUR, 75CUR:25PTX and 50CUR:50PTX formulations did not induce any significant G2/M arrest compared to the untreated cells. Consistent with the MTS assay, the treatment of Beas-2B with PTX caused a significant increase in sub-G1 populations from 7.0 ± 0.6% to 30.3 ± 5.16% (*p* < 0.0001) (Figure 6). Several studies have demonstrated that CUR and PTX inhibited the proliferation of cancer cells by interrupting the cell cycle process [9,35]. Specifically, PTX inhibits the replication transition from G0 to G1 and induces G2/M arrest [9]. Meanwhile, CUR is involved in the inhibition of the premitotic G2 and mitotic phase of the cell cycle. CUR also acts to induce apoptosis/necrosis in cells, such as the tumor suppressor p53 [36]. In our cell cycle data, CUR and PTX, either administered alone or in combination, consistently inhibited cell proliferation via the induction of G2/M arrest leading to apoptotic/necrotic death.

**Figure 7:**
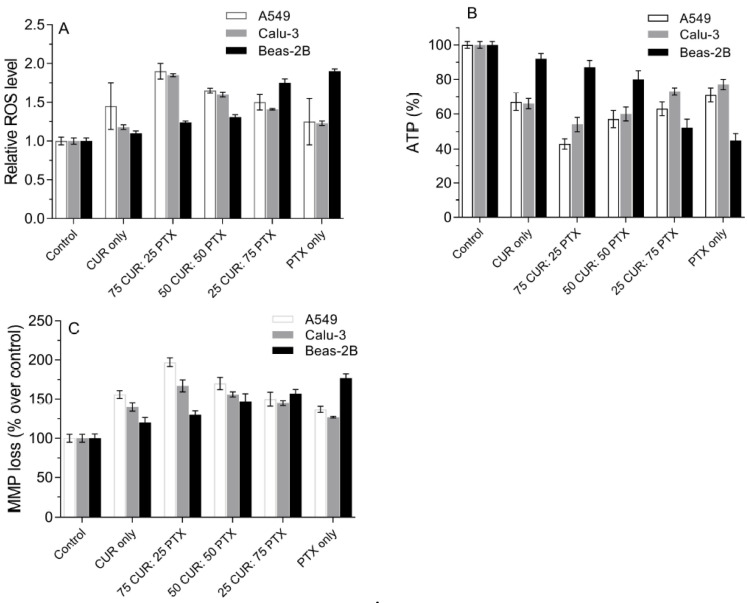
Effect of CUR, PTX and CUR/PTX combination formulations on the (**A**) intracellular ROS level, (**B**) ATP levels and (**C**) MMP depletion in A549, Calu-3 and Beas-2B. Control indicates untreated cells. Data are expressed as mean ± SD of three replicates.

Reactive oxygen species is one of the most important mediators in modulating the proliferation and protein signaling in eukaryotic cells. However, excessive ROS in the cell’s body could induce a toxicity effect which leads to cell death. This has become an important tool in cancer treatment, as most chemotherapeutic agents possess the ability to induce ROS in cancer cells. It has been reported that CUR and PTX promoted ROS accumulation in a wide range of cancerous cells, which subsequently results in cell death. In this study, we attempted to measure the amount of ROS generated from A549, Calu-3 and Beas-2B cells in response to treatment with CUR, PTX and CUR/PTX combination formulations, using a fluorescent probe (DCFH-DA). The result from Figure 7A demonstrates that the production of ROS in three lung cell lines is significantly higher than in the untreated control. For A549 and Calu-3, treatment with CUR alone resulted in a higher relative ROS level compared to PTX only. However, the production of ROS in A549 is higher compared to Calu-3 when treated with CUR alone or PTX alone. The relative ROS increase, when treated with CUR alone, is 1.45 for A549 cells as opposed to 1.18 for Calu-3 cells. It should be noted the ROS level is compared to the control, in which the value is standardized to 1. The reduction in ROS level in lung cancer cells when treated with PTX is associated with the uncoupling protein 2 that is located in the inner membrane of the cell mitochondria [37]. Our study showed that the combination of CUR and PTX formulations resulted in significantly higher ROS levels compared to CUR alone or PTX alone. As expected, the 75CUR:25PTX treatment resulted in the highest ROS production in both A549 and Calu-3 cells. A reduction in CUR ratio in the combination formulation was accompanied with significantly reduced ROS level. As seen in Figure 7, the relative ROS levels dropped from 1.9 ± 0.1 to 1.5 ± 0.1 and 1.85 ± 0.02 to 1.41 ± 0.01 for A549 and Calu-3, respectively. Our data are in agreement with other published findings, which reported the synergism of PTX and CUR in promoting ROS accumulation within cells [38]. Muthoosamy et al. demonstrated that the dual loading of PTX and CUR onto the polymer-functionalized graphene oxide resulted in the highest accumulation of ROS in A549 and MDA-MB-231 [39]. The introduction of 5–10 µM of CUR into PTX (25 nM) induced a significantly higher production of ROS in C6 glioma cells [40]. Based on Figure 7A, the production of ROS level in Beas-2B cells is elevated with the increment of the PTX portion in the combination formulation. A relative increase of up to 1.9 in ROS level was noted when cells were treated with PTX alone. The relative ROS level followed this decreasing pattern: PTX only (1.9-fold over control) > 25 CUR:75 PTX (1.75-fold over control) > 50 CUR:50 PTX (1.31-fold over control) > 75 CUR:25 PTX (1.24-fold over control) > CUR only (1.1-fold over control) (Figure 7A). We speculated that the increase in ROS levels correlated with the toxicity of the drugs. Specifically, the oxidative stress is induced mainly by PTX, as CUR is non-toxic to Beas-2B. Chen et al. also reported that flavonoid induced insignificant amounts of ROS and pro-apoptotic effects in Beas-2B cells [41].

A sufficient amount of energy is important to maintain the biological activities in mammalian cells. Compared to healthy cells, cancerous cells generally require more energy production from the mitochondria, thus it would be interesting to evaluate the production of ATP in A549 and Calu-3 cells after they are treated with a different combination of the CUR and PTX formulation (Figure 7B). Our results demonstrated that both cell lines produced significantly less ATP after being treated with CUR or PTX alone and the CUR/PTX combination, as compared to the untreated cells (control). The ATP level is correlated to the concentration of CUR present in the formulation. In general, the higher the ratio of CUR to PTX, the higher the reduction in ATP level in cancer cells (Figure 7B). The ATP level of A549 was reduced to 43% when treated with the 75CUR:25PTX formulation. Meanwhile, for the 25CUR:75PTX formulation, the ATP production was 63.0 ± 4%, indicating that the viability of cells, when treated with this formulation, is higher. A similar ATP production profile is also observed with Calu-3 cells when treated with the CUR/PTX combination at varying ratios. The ATP productions ranged from 54 to 77% (Figure 7B). The results showed that the formulations are more effective in targeting A549 cells compared to Calu-3 cells. For instance, when cells were treated with the 25CUR:75PTX formulation, the ATP values for A549 and Calu-3 cells were 63 ± 4% and 73 ± 2%, respectively. For Beas-2B cells, the ATP production is in line with the cytotoxicity of the drug formulations, whereby a formulation with high PTX fraction resulted in a higher reduction of ATP level. For instance, the ATP level in Beas-2B reduced to 45 ± 4% when treated with PTX alone. Meanwhile, for a combination formulation of 25CUR:75PTX and 75CUR:25PTX, the ATP levels were 52 ± 5% and 87 ± 4%, respectively (Figure 7B). Several studies have correlated the ATP levels and the survival of drug-resistant cancer cells [42,43]. The blockage of the glycolysis pathway with 2-deoxyglucose and 3-bromopyruvate in HCT116 colon cancer cells effectively reduced intracellular ATP production and subsequently induced cell death [42]. Recently, Siddiqui et al. demonstrated that CUR inhibits aerobic glycolysis and the Warburg effect in lung cancer, while exerting negligible effects over healthy cells [44]. The Warburg effect with aerobic glycolysis is associated with high glucose uptake and efficient ATP synthesis to support the rapid proliferation of cancer cells. The authors found that a low concentration of CUR was able to induce the inhibition of metabolism, as was evident from the low glucose consumption and lactate production. Consequently, the growth of cancer cells was hampered [44,45].

The metabolic reprogramming in cancer cells often leads to the hyperpolarization of the mitochondrial membrane to accommodate the higher ATP demand for rapid cell proliferation and tumor progression [46,47]. From the cancer treatment perspective, the effectiveness of chemotherapeutic drugs could be determined by measuring the mitochondrial dysfunction using mitochondrial membrane potential (MMP). The reduction in MMP in cancer cells, apoptosis, and cytochrome C release into cytosol are indicators of mitochondrial dysfunctions [48]. As is shown in Figure 7C, all formulations resulted in the depletion of MMP in A549, Calu-3 and Beas-2B cells. As PTX is inherently toxic towards Beas-2B, it is anticipated that the MMP loss in Beas-2B would be a few magnitudes higher compared to CUR. As expected, the percentage of MMP loss over control in Beas-2B for CUR alone (120 ± 7%) is significantly lower than PTX alone (177 ± 5%), which is an indication of lower cells depolarization and toxicity. Instead, CUR alone is more able to promote MMP depolarization in both A549 and Calu-3 compared to PTX alone. These findings corroborated with the MTS and apoptosis results, which suggested CUR/PTX-induced mitochondrial dysfunction. Jung et al. reported that CUR caused a dose-dependent reduction in MMP coupled with suppressed mitochondrial oxidative respiration [49]. Many studies have observed that CUR is responsible for the damage of mitochondria, which leads to increased proton leak, the depolarization of MMP and the subsequent initiation of apoptosis [49,50]. As expected, the combination formulation with the highest CUR ratio demonstrated the highest MMP loss in both A549 (197 ± 6%) and Calu-3 (167 ± 8%). Although the reduction in CUR ratio in the combination formulation resulted in reduced MMP loss, it was still more effective than CUR and PTX alone (Figure 7C). Hossain et al. showed that the dual treatment of PTX and CUR induced a higher mitochondrial release of cytochrome C from human glioblastoma cell lines (LN18 and U138MG) [51]. In another study, the co-loading of PTX and CUR onto graphene oxide also provided a synergistic effect on MMP depletion in A549 and MDA-MB-231 cells [39].

## 4. Conclusions

This study reported a simple DPI formulation of a CUR and PTX combination prepared using the co-milling process. The CUR/PTX combination formulation showed suitable aerosol performance, as demonstrated in the high FPF values (60–70%) and appropriate MMAD (2.64–3.12 µm) values. The combination of PTX and CUR demonstrated a stronger anti-cancer effect in inhibiting cell proliferation, an anti-apoptotic effect, and it initiated cell cycle arrests in lung cancer cells. The efficacy is more pronounced as the ratio of CUR is increased in the formulation. In addition, the increase in ROS level and mitochondrial depolarization indicate that the actions of CUR and PTX were associated with the regulation of mitochondrial-associated oxidative stress. Interestingly, the presence of CUR is crucial in mitigating the cytotoxic effects of the chemotherapeutic agent against healthy cells. Our results demonstrated that CUR conferred cytoprotective effects towards Beas-2B, and the activity was dose-dependent. A combination formulation of a chemotherapeutic agent with a chemoprotective agent such as CUR is an interesting concept to enhance anticancer activities towards cancer cells, while simultaneously protecting healthy cells from irreversible damages.

## Figures and Tables

**Table 1:** Particle size distribution of milled formulations of CUR, PTX and CUR/PTX. CUR: curcumin; PTX: paclitaxel.

Formulations/ Ratio	Particle Size (µm ± StDev)		
	d_10_	d_50_	d_90_
CUR only	1.0 ± 0.1	2.7 ± 0.4	5.2 ± 0.7
75CUR:25PTX	1.5 ± 0.2	2.2 ± 0.3	4.9 ± 0.3
50CUR:50PTX	0.9 ± 0.1	3.3 ± 0.5	5.4 ± 0.4
25CUR:75PTX	1.2 ± 0.2	3.1 ± 0.5	5.1 ± 0.8
PTX only	1.3 ± 0.1	3.7 ± 0.4	5.7 ± 0.6

**Table 2:** FPF, MMAD, ED and FPD of DPI formulations of CUR and PTX using the RS01 HR device.

Formulation	CUR Only	75CUR:25PTX		50CUR:50PTX		25CUR:75PTX		PTX Only
		CUR	PTX	CUR	PTX	CUR	PTX	
FPF (%)	69.5 ± 1.0	64.7 ± 7.8	69.6 ± 3.5	65.6 ± 2.2	59.8 ± 4.5	55.8 ± 7.0	53.1 ± 5.9	60.4 ± 1.5
MMAD (µm)	3.00 ± 0.05	3.12 ± 0.04	2.74 ± 0.05	2.64 ± 0.09	2.78 ± 0.05	2.82 ± 0.12	2.89 ± 0.13	2.85 ± 0.10
ED (µg)	6392.6 ± 254.6	4887.6 ± 195.0	1755.8 ± 37.0	3557.0 ± 150.0	3429.4 ± 140.4	1600.0 ± 40.0	5263.0 ± 160.0	6044.2 ± 184.2
FPD_EM_ (µg)	4442.86 ± 64.0	3162.3 ± 381.2	1235.9 ± 62.1	2333.3 ± 78.2	2050.8 ± 154.2	892.8 ± 112	2794.7 ± 310.3	3650.7 ± 91.0

n = 3, mean ± SD; FPF: fine particle fraction; MMAD: mass median aerodynamic diameter; ED: emitted dose; FPD_EM:_ fine particle emitted dose.

**Table 3:** IC_50_ values of single and combination formulations assayed using MTS cytotoxicity.

Cell Line	IC_50_ (µM)				
	CUR Only	75CUR:25 PTX	50CUR:50PTX	25CUR:75PTX	PTX Only
A549	26.3 ± 2.9 ^c^	18.9 ± 3.5 ^b^	22.1 ± 0.5 ^b^	32.5 ± 2.3 ^d^	47.5 ± 2.6 ^f^
Calu-3	30.3 ± 2.5 ^c,d^	22.9 ± 1.8 ^b^	23.1 ± 2.5 ^b^	29.1 ± 1.5 ^c,d^	39.5 ± 1.6 ^e^
Beas-2B	ND	ND	ND	20.3 ± 1.7 ^b^	8.1 ± 0.5 ^a^

ND: not detected at the tested concentration range. Values (mean ± SD of three replicates) in the same row and not having the same superscript are significantly different at *p* < 0.05 (Tukey post hoc test).

## Data Availability

Not applicable.
